# Identification of TNFRSF21 as an inhibitory factor of osteosarcoma based on a necroptosis-related prognostic gene signature and molecular experiments

**DOI:** 10.1186/s12935-023-03198-w

**Published:** 2024-01-06

**Authors:** Xiang Li, Zhenqian Sun, Jinlong Ma, Miaomiao Yang, Hongxin Cao, Guangjun Jiao

**Affiliations:** 1 Department of Orthopedics, Qilu Hospital of Shandong University, No.107, Wenhuaxi Road, Lixia District, Jinan, 250000 Shandong Province China; 2 Cheeloo College of Medicine, Shandong University, Jinan, Shandong Province China; 3 Department of Oncology, Yantai Yuhuangding Hospital, Yantai, Shandong Province China; 4 Department of Medical Oncology, Qilu Hospital of Shandong University, Jinan, Shandong Province China; 5 Key Laboratory of Chemical Biology (Ministry of Education), Institute of Biochemical and Biotechnological Drug, School of Pharmaceutical Sciences, Cheeloo College of Medicine, Shandong University, Jinan, Shandong Province China

**Keywords:** Necroptosis, Prognostic signature, Osteosarcoma, TNFRSF21

## Abstract

**Background:**

Osteosarcoma is one of the most common malignant bone tumors with bad prognosis. Necroptosis is a form of programmed cell death. Recent studies showed that targeting necroptosis was a new promising approach for tumor therapy. This study aimed to establish a necroptosis-related gene signature to evaluated prognosis and explore the relationship between necroptosis and osteosarcoma.

**Methods:**

Data from The Cancer Genome Atlas was used for developing the signature and the derived necroptosis score (NS). Data from Gene Expression Omnibus served as validation. Principal component analysis (PCA), Cox regression, receiver operating characteristic (ROC) curves and Kaplan-Meier survival analysis were used to assess the performance of signature. The association between the NS and osteosarcoma was analyzed via gene set enrichment analysis, gene set variation analysis and Pearson test. Single-cell data was used for further exploration. Among the genes that constituted the signature, the role of TNFRSF21 in osteosarcoma was unclear. Molecular experiments were used to explore TNFRSF21 function.

**Results:**

Our data revealed that lower NS indicated more active necroptosis in osteosarcoma. Patients with lower NS had a better prognosis. PCA and ROC curves demonstrated NS was effective to predict prognosis. NS was negatively associated with immune infiltration levels and tumor microenvironment scores and positively associated with tumor purity and stemness index. Single-cell data showed necroptosis heterogeneity in osteosarcoma. The cell communication pattern of malignant cells with high NS was positively correlated with tumor progression. The expression of TNFRSF21 was down-regulated in osteosarcoma cell lines. Overexpression of TNFRSF21 inhibited proliferation and motility of osteosarcoma cells. Mechanically, TNFRSF21 upregulated the phosphorylation levels of RIPK1, RIPK3 and MLKL to promote necroptosis in osteosarcoma.

**Conclusions:**

The necroptosis prognostic signature and NS established in this study could be used as an independent prognostic factor, TNFRSF21 may be a necroptosis target in osteosarcoma therapy.

**Supplementary Information:**

The online version contains supplementary material available at 10.1186/s12935-023-03198-w.

## Introduction

Osteosarcoma is the most common primary bone malignancy with high aggressiveness [1, 2]. Despite advances in osteosarcoma management, which consists of surgical excision, chemotherapy, radiotherapy and immunotherapy, the 5-year survival rate of patients with metastasis is less than 20% [3]. Thus, curbing of metastasis and recurrence remains a serious problem [4], and looking for new targets might be breakthrough for osteosarcoma therapy.

Necroptosis, a newly recognized programmed cell death (PCD), has attracted great attention in recent years. Receptor interacting serine/threonine kinase 1 (RIPK1), RIPK3, and mixed lineage kinase domain-like (MLKL) were core mediators of necroptosis [5]. Mechanistically, phosphorylative activation of RIPK1 at the S166 site is the first event in necroptosis process. And RIPK3 (at the S227 site) and MLKL (at the S358 site) will be phosphorylated subsequently and sequentially [6]. Finally, phosphorylated MLKL will cause formation of oligomers that punctures the plasma membrane and causes subsequent cell death [7]. Morphologically, necroptosis is characterized by membrane rupture and organelle swelling [8].

Currently, it is believed that necroptosis is an important event in tumor development and progression. Marco Seehawer et al. found that hepatocytes with aberrantly activated oncogenes (TBX3 and PRDM5) gave rise to cholangiocarcinoma, when embedded in a necroptosis-dominated hepatic microenvironment [9]. There was also evidence that RIPK3 restricted myeloid leukemogenesis by promoting cell death [10]. In addition, necroptosis is also involved in tumor immune response [11]. For example, dying cancer cells with deficiency of RIP3 or MLKL cannot elicit an immune response [12]. However, few literatures have referred on necroptosis in osteosarcoma. Suoyuan Li et al. used shikonin to upregulate necroptosis in osteosarcoma, which led to a reduction in pulmonary metastasis [13]. Their work indicated the important role of necroptosis in osteosarcoma development. One of the most critical points in tumor therapy is drug resistance (such as venetoclax) induced by the evasion or resistance of tumor cell to PCD [14–16]. Taking this one step further, direct promotion of necroptosis to the tumor cells themselves will be a more potential therapeutic way.

It has been reported that tumor necrosis factor receptor superfamily member 21 (TNFRSF21) induced cell apoptosis in the nervous system after binding to cleaved amino-terminal fragment of APP [17]. Strilic et al. found that TNFRSF21 bound to APP activated by tumor cells and caused necroptosis but not apoptosis in vascular endothelial cells [18]. TNFRSF21 may induce other types of PCD in tumor [19]. TNFRSF21, activated by α-ketoglutarate, could induce caspase 8 to gasdermin C leading to pyroptosis. In the process, APP was not even involved. However, the role of TNFRSF21 and the exactly mechanisms in mediating PCD in osteosarcoma is still not clarified.

In this study, necroptosis-related genes (NRGs) associated with osteosarcoma prognosis were screened to construct the necroptosis prognostic signature and the derived necroptosis score (NS). And the underlying mechanism of TNFRSF21 in osteosarcoma was further investigated.

## Methods and materials

### Data collection

Clinical information and bulk RNA-sequencing data of osteosarcoma were downloaded from The Cancer Genome Atlas (TCGA) and set as the training cohort (n = 84). The pertinent information is presented in Supplemental Table 1. GSE39055 (Platform: GPL14951 Illumina HumanHT-12 WG-DASL V4.0 R2 expression beadchip. Public on 2013) and GSE21257 (Platform: GPL10295 Illumina human-6 v2.0 expression beadchip. Public on 2011) were downloaded from the Gene Expression Omnibus (GEO). For datasets, patients with unknown survival status will be excluded. After the adjustment for batches, the two datasets were then merged as validation cohort (n = 89) [20].

### Construction and validation of the necroptosis-related prognostic gene signature

NRGs were retrieved from GeneCards. Prognostic genes were firstly identified by univariate Cox regression. The hub genes were further screened and confirmed by the least absolute shrinkage and selection operator (LASSO). The NS was calculated as followed: NS = ${\sum }_{\text{i}}^{\text{n}}\text{Exp}\text{i}\text{Coe}\text{i}$ (Exp = expression level; Coe = regression coefficient).

Principal component analysis (PCA), Cox regression and time-dependent receiver operating characteristic (ROC) curves were applied to evaluate the performance of the signature. According to the median NS in the training cohort, patients were divided into high- and low-score groups. Kaplan-Meier (KM) survival analysis was performed to compare survival rates between two groups.

Finally, gender, age and NS were combined to create a nomogram to assess prognosis (overall survival rate) of osteosarcoma patients. Calibration curves and decision curve analysis were applied to evaluate the nomogram.

### Functional analysis in TCGA cohort

Compared to the low-score group, the differentially expressed genes (DEGs) in high-score group were identified using the ‘limma’ R package [21]. When the DEGs were obtained, false discovery rate (FDR) < 0.05 and |log_2_FC| > 1 was considered statistically significant. Enrichment analysis in Gene Ontology (GO) and Kyoto Encyclopedia of Genes and Genomes (KEGG) was performed with ‘ClusterProfiler’ R package [22]. Gene set enrichment analysis (GSEA) and gene set variation analysis (GSVA) were used to explore the alterations in biological function between two groups [23, 24]. The preset gene set was GOBP necroptotic signaling pathway and h.all.v7.5.1.symbols. | normalized enrichment score (NES)| > 1, nominal *P* < 0.05 and FDR q < 0.25 were considered statistically significant. The activity of apoptosis and necroptosis signal pathways in high-score group was visualized using the ‘pathview’ R package [25].

Immune infiltration levels were calculated by single sample gene set enrichment analysis (ssGSEA) [26]. Microenvironment scores and tumor purity of osteosarcoma were calculated by ‘ESTIMATE’ R package [27]. Stemness indices were calculated by one-class logistic regression (OCLR) [28]. Pearson correlation coefficient was used to assess the correlations between these parameters and NS.

Osteosarcoma samples were divided into two groups based on the median expression of gene. Then the effect of single gene was evaluated by KM survival analysis. Subgroup analysis was performed to assess whether the expression of TNFRSF21 and necroptosis mediators were associated with clinical characteristics. Finally, we calculated the Pearson correlation coefficient of TNFRSF21 and necroptosis mediators.

### Single-cell data analysis

Osteosarcoma single-cell data (GSE162454) was collected from GEO and then imported into ‘Seurat’ R package with default parameters [29]. For count matrix in the dataset, a standard pipeline in ‘MAESTRO’ in TISCH website was used to perform quality control, clustering and cell-type annotation [30, 31].

According to the formula, we calculated the NS of each cell and average NS of each cell population. Malignant cells with the top 5% and bottom 5% NS were assigned to the high-NS and low-NS groups, respectively. The count matrix was imported in to ‘CellChat’ R package to investigate cell–cell interactions between two groups [32].

### Cell culture and lentivirus infection

Human osteoblast cell line hFOB1.19 (RRID: CVCL_3708) and three human osteosarcoma cell lines HOS (RRID: CVCL_0312), MG63 (RRID: CVCL_0426) and U2OS (RRID: CVCL_0042) were used in this study. Osteosarcoma cell lines were purchased from Zhong Qiao Xin Zhou Biotechnology Co., Ltd. (Shanghai, China), hFOB1.19 was purchased from Procell Biotechnology Co., Ltd. (Wuhan, China). Before experiments, DNA types of cell lines were identified by short tandem repeat (STR) profiling and compared with DSMZ data. All experiments were performed with mycoplasma-free cells which were cultured according to the instructions.

Lentivirus containing pLVX-TNFRSF21-Puro and pLVX-Puro (vector) were purchased from Genechem (Shanghai, China) and used to infect HOS and MG63 according to the manufacturer’s protocol. Stably transfected cells were selected with puromycin and validated by polymerase chain reaction (PCR) and western blot.

### Western blot and PCR

Cells were lysed using RIPA buffer (Cell Signaling Technology, Danvers, MA, United States). Western blot was performed according to previous study [33]. B-actin (1:10000) was set as control. Other antibodies used were: TNFRSF21 (1:1000), RIPK1 (1:1000), p-RIPK1 (S166) (1:1000), RIPK3 (1:1000), p-RIPK3 (S227) (1:1000), MLKL (1:1000) and p-MLKL(S358) (1:1000). All antibodies were purchased from Cell Signaling Technology (Cell Signaling Technology, Danvers, MA, United States).

Cells were lysed using TRIzol reagent (Life Technologies, CA, USA). PCR was performed to determine mRNA expression levels according to previous study [34]. B-actin was set as control and relative mRNA expression levels were calculated by the 2^–ΔΔCt^ method. The sequences of primers were included in Supplemental Table 2.

### Detection of cell proliferation and motility

For CCK-8 assays, cells were seeded at a density of 1 × 10^3^ cells/well in 96-well plate. Ten µl of CCK-8 reagent was added at specific timepoints. Optical density (OD) was measured at 450 nm after incubation at 37 °C for one hour.

For colony formation assays, cells were seeded at a density of 1 × 10^3^ cells/well in 6-well plate [35]. Medium was changed every two days. After one week, cell colonies were fixed with paraformaldehyde (4%) for 30 min and stained with crystal violet.

The fraction of DNA-replicating cells, which represented cell proliferation status, was assessed using EdU Detection Kit (RiboBio, Guangzhou, China).

Four-week-old male BALB/c nude mice were purchased from the Laboratory Animal Center of Shandong University (Jinan, China). The mice were placed in the Laboratory Animal Center of Shandong University Qilu Hospital and were fed in an air-conditioned room at 23–25 °C with a light-dark cycle time of 12 h, where they had ample access to water and food. Nude mice were injected subcutaneously with 2 × 10^6^ stably transfected HOS cells or control cells. After four weeks, all mice were sacrificed and the xenografts weighed and harvested. Tumor volume was calculated as follows: Volume (mm3) = (length × width^2^)/2 [36]. The animal experiments were approved by the Animal Ethics Committee of Qilu Hospital of Shandong University (No. DWLL-2023-028).

Transwell assays were performed according to previous study [36]. At the beginning, 1 × 10^4^ cells per group were inoculated in the upper chamber. After 24 h, cells in the bottom chamber were fixed, stained and photographed.

For wound healing assays, a sterile pipette tip was used to scratch and form a gap when cells were completely confluent in 6-well plate. Then serum-free medium was added. After incubation for 12 h, the gap was photographed.

### Apoptosis detection

Cells were seeded at a density of 1 × 10^4^ cells/well in 24-well plate [35]. TUNEL staining was performed using TUNEL BrightRed Apoptosis Detection Kit (Vazyme, China).

Cell suspensions were made in phosphate-buffered saline. Annexin V-FITC Apoptosis Detection Kit (Vazyme, China) and flow cytometry were used to detect apoptosis. Cells in the upper right quadrants (PI positive and Annexin V positive) were considered necrotic.

According to the product instruction, relative activity of caspase 8 was detected by Caspase 8 Activity Assay Kit (Beyotime Biotechnology, Shanghai, China).

### Statistical analysis

All analyses were performed in RStudio (version 4.1.2). All experiments were carried out at least three times independently. Data from experiments (such as PCR and cell proliferation assay) was investigated with two-tailed Student t test. Wilcoxon–Mann–Whitney nonparametric test was used for subgroup analysis of TNFRSF21 and necroptosis mediators in osteosarcoma. The correlation coefficients were derived from the Pearson tests. Statistical significance was set at *P* < 0.05.

## Results

### Construction and validation of the necroptosis-related prognostic gene signature

**Fig. 1:**
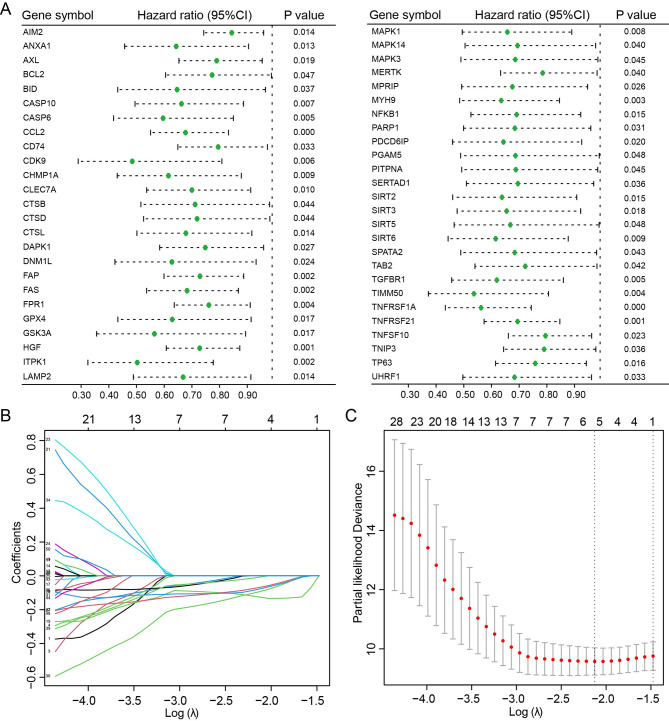
Screening of hub necroptosis-related genes from osteosarcoma. Forest plot showed all necroptosis-related genes associated with prognosis in osteosarcoma obtained by univariate Cox regression (**A**). LASSO coefficient profiles were depicted and the optimal values of the penalty parameter were defined (**B**, **C**)

611 NRGs were obtained from GeneCards. Univariate Cox regression showed hazard ratios of NRGs were < 1, which indicated necroptosis might be a favorable factor for osteosarcoma prognosis (Fig. 1A). Then five hub prognostic NRGs were obtained by LASSO (Fig. 1B, C), including CCL2, FAP, HGF, TNFRSF1A and TNFRSF21.

**Fig. 2:**
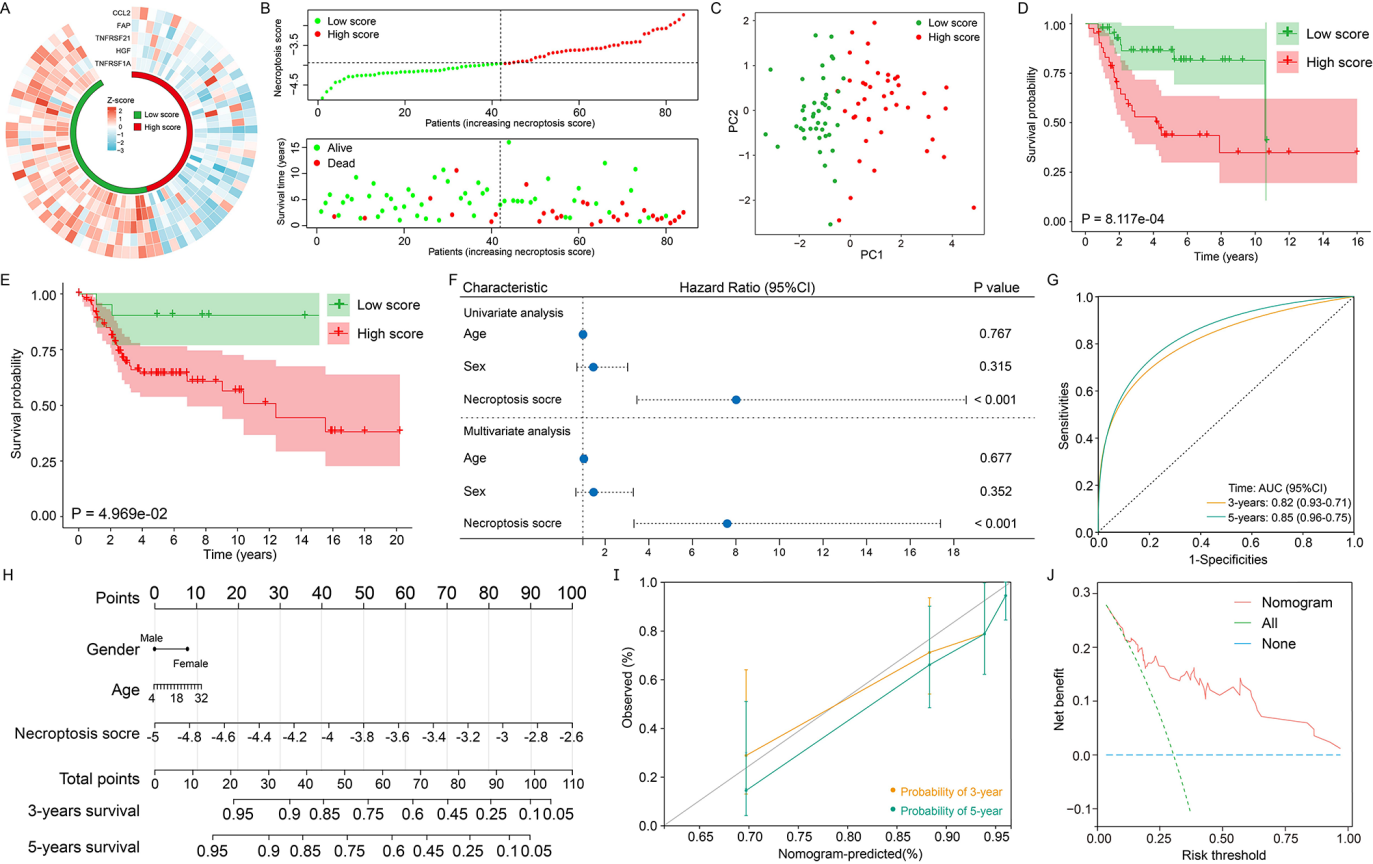
Construction and validation of the necroptosis-related prognostic gene signature. The normalized expression profiles of hub NRGs (**A**). The necroptosis score distribution and survival status of patients in the training cohort (**B**). PCA results for two groups of patients in the training cohort (**C**). Kaplan-Meier survival analysis of patients in the training cohort (**D**) and validation cohort (**E**). COX regression showed that necroptosis score was a prognostic factor independent of other clinical characteristics (**F**). Receiver operating characteristic curves of the necroptosis score score in distinguishing patient prognosis (**G**). The nomogram consisted of gender, age and necroptosis score based on the five-NRG signature (**H**). The calibration curves for internal validation of the nomogram for estimating the survival of osteosarcoma patients at 3 and 5 years (**I**). Decision curve analysis indicated a greater net benefit of nomogram (**J**)

In the training cohort, the normalized expression of five NRGs was presented in Fig. 2A. Next, the NS of each patient was calculated: NS = CCL2 × -0.078 + FAP × -0.008 + HGF × -0.068 + TNFRSF1A × -0.125 + TNFRSF21 × -0.094. Of note, lower NS indicated more active necroptosis in osteosarcoma.

As shown in Fig. 2B, patients were stratified into a high-score and low-score subgroup using the median NS as the cutoff value. PCA demonstrated that patients were effectively distinguished via NS (Fig. 2C). KM survival analysis showed patients in high-score group had worse prognosis (*P* < 0.001) (Fig. 2D). In the validation cohort, patients in high-score group showed significantly inferior survival rates than low-score patients (*P* < 0.05) (Fig. 2E). Moreover, NS was an independent prognostic factor (Fig. 2F). In Fig. 2G, the area under the curve was 0.82 in 3-year and 0.85 in 5-year. In summary, these data demonstrated the satisfactory accuracy and generalizability of the signature based on five NRGs.

Finally, we developed a nomogram including gender, age and NS based on the training cohort (Fig. 2H). In Fig. 2I, calibration curves showed the predicted probability of 3- and 5-year overall survival was consistent with the actuality. In addition, the nomogram brought a greater net benefit for patients (Fig. 2J).

### Pathway enrichment and tumor microenvironment analysis

**Fig. 3:**
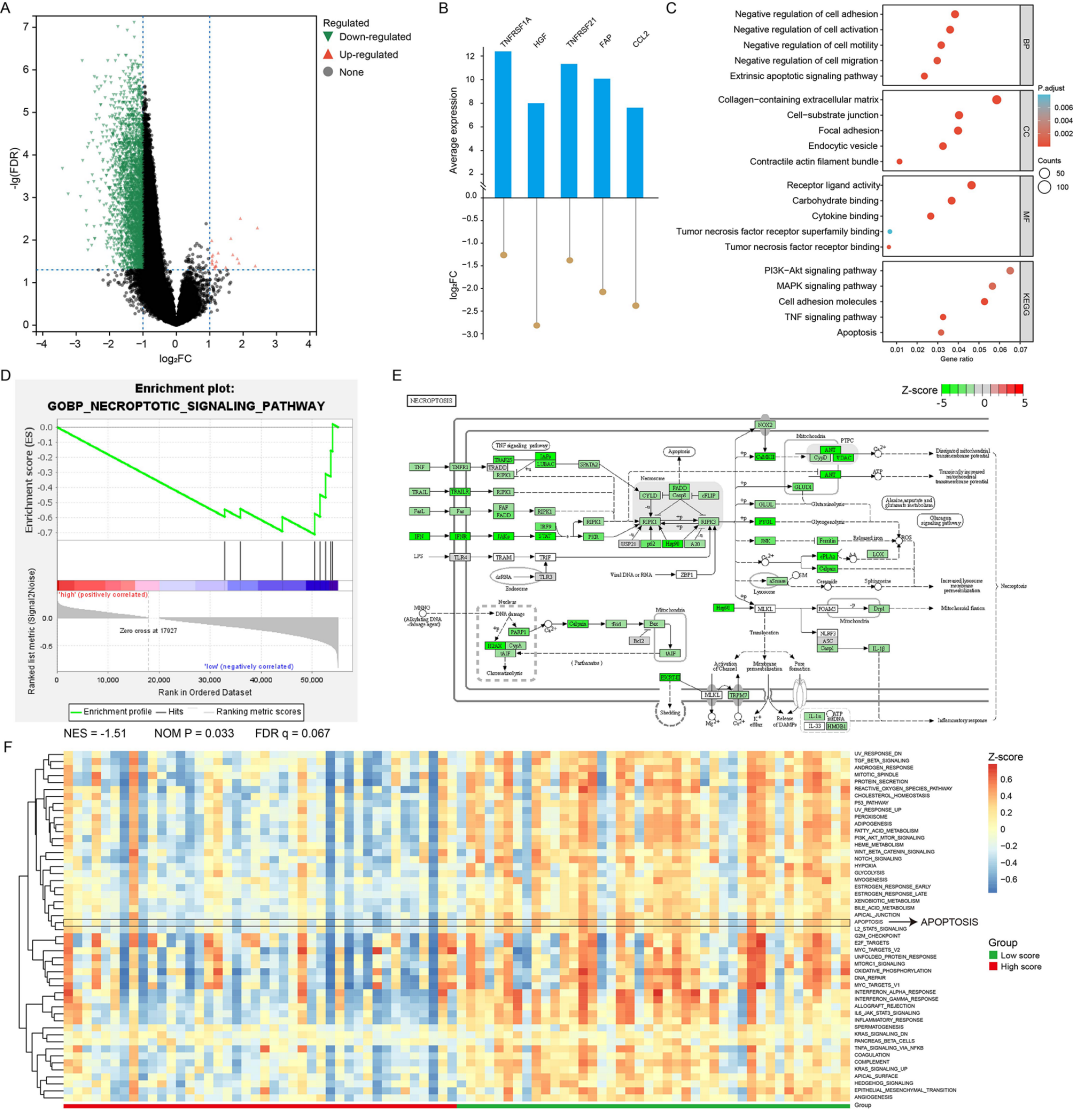
Functional analysis in TCGA cohort. Volcano map of differentially expressed genes (DEGs) in high-score group (**A**). Average expression and log2FC of five hub NRGs (**B**). Enriched functions and pathways of DEGs (**C**). GSEA result on necroptotic signaling pathway (**D**). The pathway plot indicated that necroptosis was inhibited in high-score group (**E**). Heatmap illustrating the results of GSVA (**F**)

After calculation, we obtained DEGs in the high-score group, which included NRGs that constructed the signature (Fig. 3A). As in Fig. 3B, these five NRGs were down-regulated, which indicated that necroptosis was inhibited in the high-score group.

Next, enrichment analysis of DEGs was performed to explore the biological process and signal pathways that related to NS. In Fig. 3C, DEGs were involved in PI3K-Akt signaling pathway and MAPK signaling pathway. In the terms of molecular function, DEGs was associated with receptor ligand activity. In addition, DEGs was also involved in extrinsic apoptotic signaling pathway. GSEA indicated the NES of necroptotic signaling pathway was lower in the high-score group (Fig. 3D). Pathway plot demonstrated that the processes of necroptosis were down-regulated in the high-score group (Fig. 3E). Furthermore, we also noticed that TNF signaling pathway and IFN signaling pathway were both de-activated, which meant anti-tumor immune response might be inhibited. In Fig. 3F, GSVA revealed that apoptosis process was down-regulated in the high-score group.

**Fig. 4:**
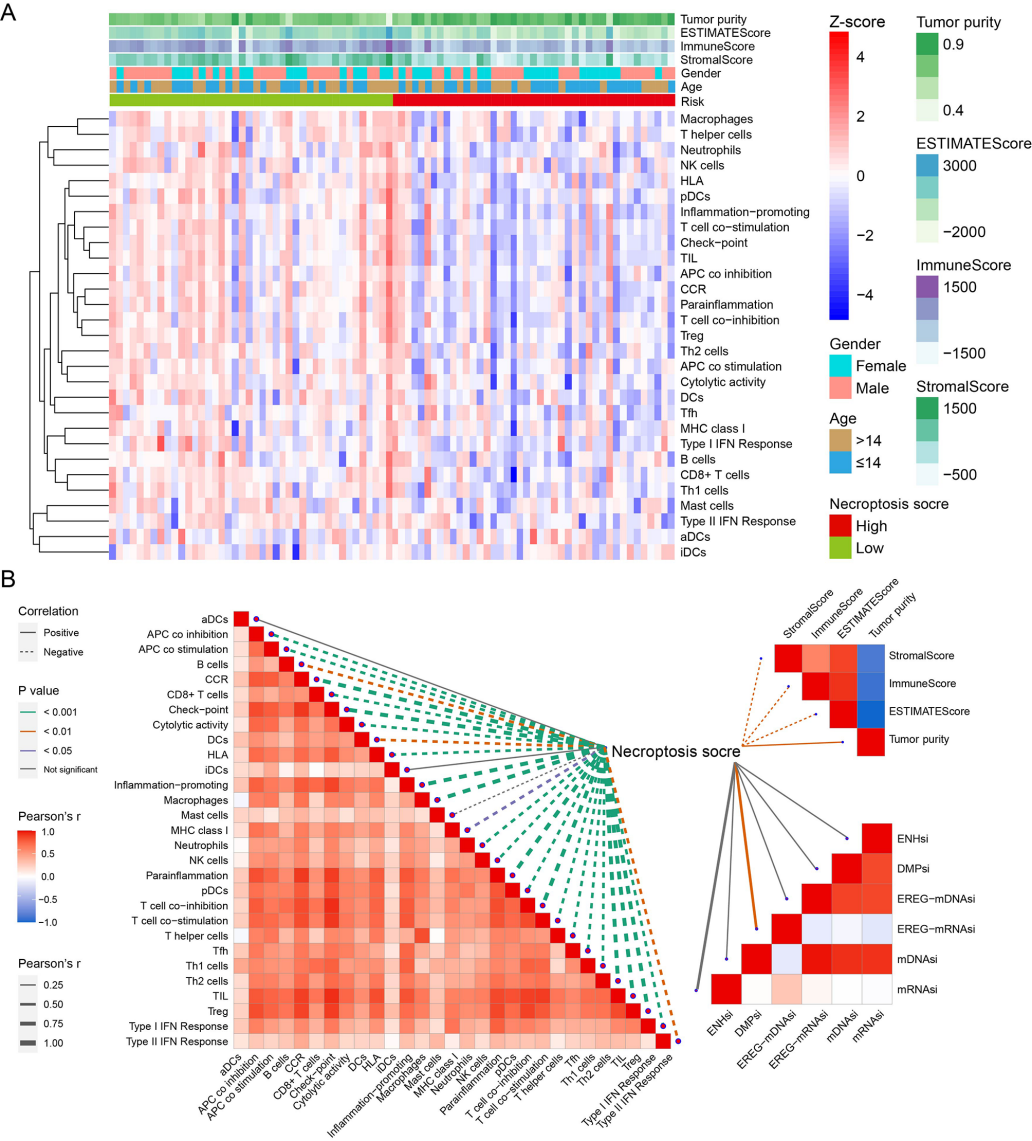
Analysis of tumor microenvironment. The profile of immune infiltration, microenvironment score and tumor purity in TCGA cohort (**A**). The Pearson correlation analysis of necroptosis score and other parameters of microenvironment (**B**)

In Fig. 4A, we demonstrated the profile of immune infiltration, microenvironment score and tumor purity in osteosarcoma. As shown in Fig. 4B, most of immune cell infiltration levels were decreased with increased NS. Since lower NS indicated more activated necroptosis, this finding meant the activity of necroptosis was positively associated with immune cell infiltration levels in osteosarcoma. The negative correlation between NS and immune score further validated our speculation. Moreover, NS was positively associated with tumor purity. This phenomenon might be caused by the EREG-mRNAsi that increased with higher NS. Because the higher the EREG-mRNAsi, the lower the tumor differentiation degree [28].

### Single-cell transcriptomics reveal necroptosis heterogeneity in osteosarcoma

**Fig. 5:**
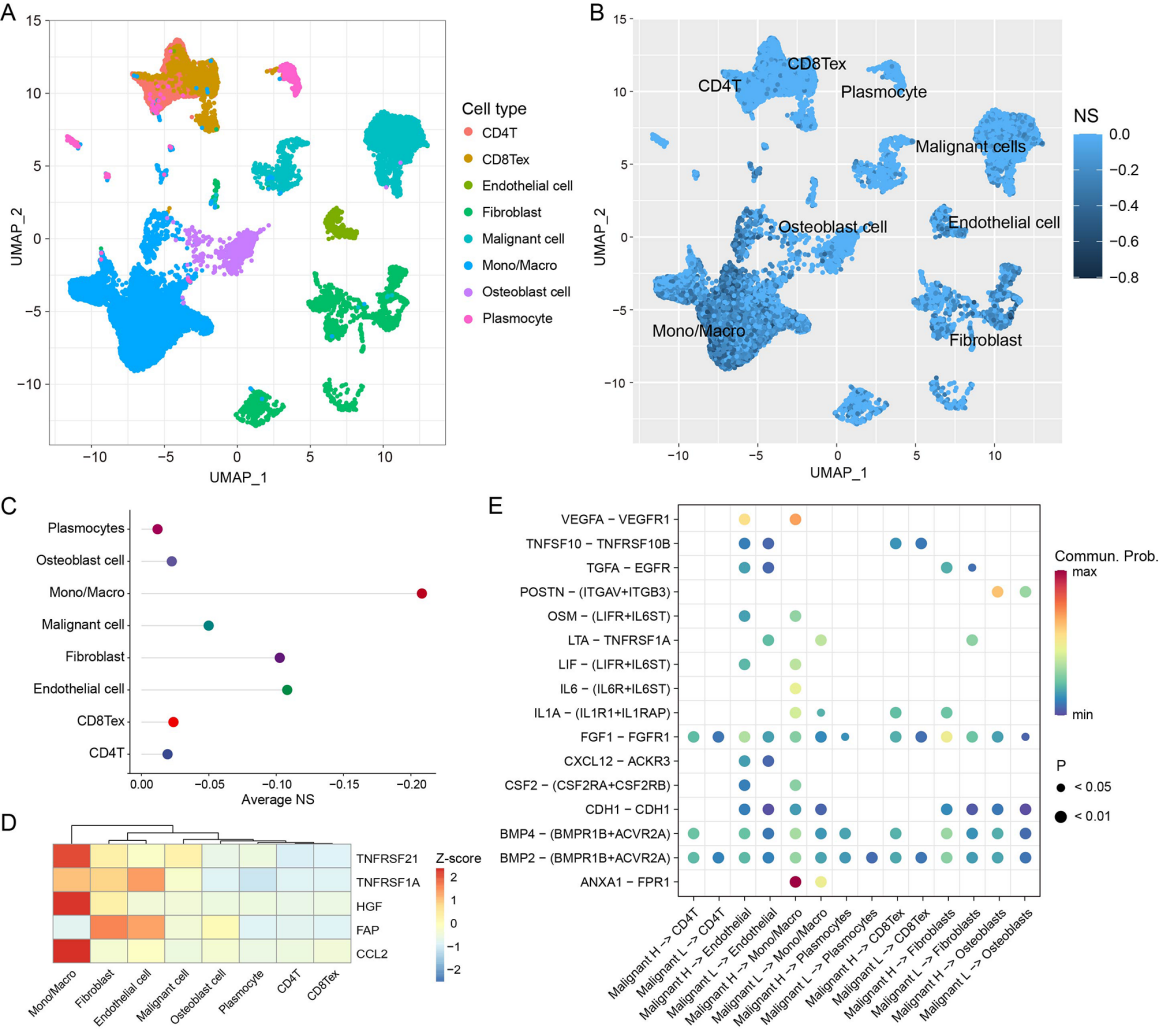
Analysis of single-cell data of osteosarcoma. Uniform manifold approximation and projection plots show eight main cell types in single-cell data (GSE162454), colored by cell type (**A**) and the NS (**B**). The average NS (**C**) and normalized expression of hub gene (**D**) in eight main cell types. Bubble chart showing significant ligand–receptor interactions between high-NS/low-NS malignant cells (malignant H/malignant L) and neighboring cells. Ligand-expressed cells and receptor-expressed cells are shown on the x-axis, ligands and receptors are shown on the y-axis. The color indicates the average expression levels of ligands and receptors in interacting cells, and the bubble size indicates the significance of the interactions (**E**)

To explore the impact of necroptosis on the tumor microenvironment, we further analyzed singe-cell data from osteosarcoma. After quality control, 46,544 cells were obtained from osteosarcoma. As shown in Fig. 5A, we identified these cells to 8 cell types: CD4^+^T cell (CD4^+^T), exhausted CD8^+^T cell (CD8^+^Tex), endothelial cell, fibroblast, malignant cells, monocyte or macrophage (Mono/Macro), osteoblast cell and plasmocyte.

Since expression of hub genes was not detected in some cells, we calculated NSs for 22,563 cells and average NSs for 8 cell types. Consistently, we observed intercellular heterogeneity of NS in the osteosarcoma microenvironment (Fig. 5B, C). In addition, the expression of hub genes in each cell type was presented (Fig. 5D). We found the expression of hub genes was higher in Mono/Macro, fibroblast and endothelial cells.

Recent findings reported that apoptotic cells had impact on the behaviors of surrounding cells. And these interactions can be bidirectional [37, 38]. We next focused on cell-cell interactions via ‘CellChat’. The data showed that malignant cells in the high- and low-NS groups had different communication patterns with neighboring cells (Fig. 5E). High-NS malignant cells showed a stronger pattern of interaction pairs for vascular endothelial growth and fibroblast growth (VEGFA-VEGFR1 and FGF1-FGFR1). For the interaction pair POSTN-(ITGAV + ITGB3), high-score malignant cells exhibited higher communication intensity. This pair is known to maintain the stemness of tumor stem cells and promote metastasis [39]. In summary, these data partially explained the poorer prognosis of patients in the high-score group and also further validate our findings from the bulk RNA-sequencing data.

### Differential expression of five NRGs and necroptosis mediators

**Fig. 6:**
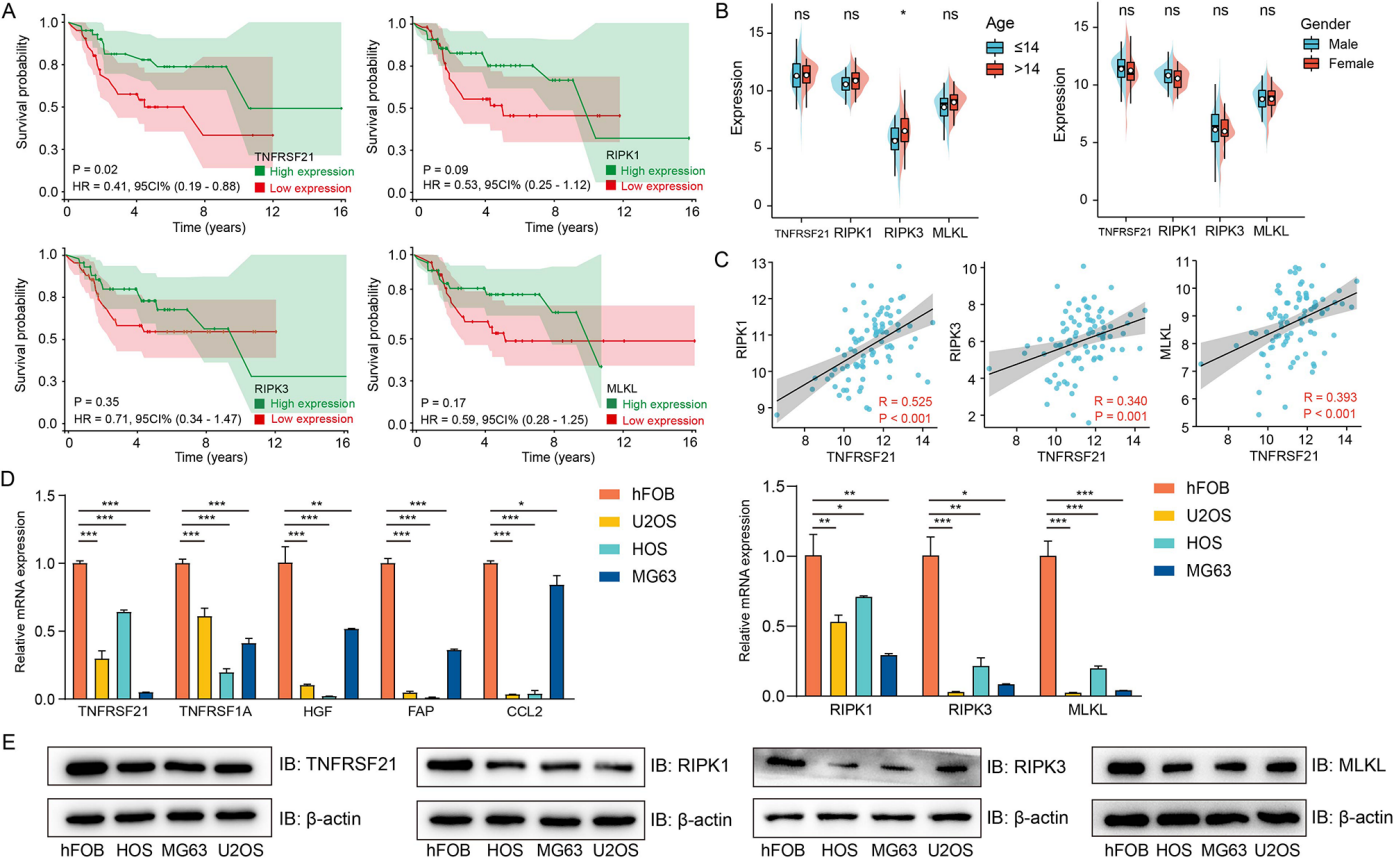
Survival analysis and expression of TNFRSF21 and necroptosis mediators in osteosarcoma. Survival analysis of single gene (**A**). Subgroup analysis, *P* value was calculated by Wilcoxon–Mann–Whitney nonparametric tests (2-tailed) (*, *P* < 0.05) (**B**). Pearson correlation analysis of TNFRSF21 and necroptosis mediators (**C**). Compared to osteoblast cell line, the expression of five hub NRGs and necroptosis mediators was down-regulated, *P* value was calculated by 2-tailed Student t test (n = 3)(*, *P* < 0.05; **, *P* < 0.01; ***, *P* < 0.001;) (**D**, **E**)

KM survival analysis revealed patients with high expression of TNFRSF21 had better prognostic, But RIPK1, RIPK3 and MLKL had no impact on patients’ survival (Fig. 6A). Stratified analysis showed the expression of TNFRSF21 and necroptosis mediators was not almost associated with age and gender (Fig. 6B). In addition, TNFRSF21 expression was positively correlated with necroptosis mediators, suggesting TNFRSF21 might promote necroptosis in osteosarcoma with better prognosis (Fig. 6C).

The expression of five NRGs and necroptosis mediators in human osteosarcoma cell lines was examined to validate the data. Compared to osteoblast cell line (hFOB1.19), mRNA expression of five NRGs and necroptosis mediators in osteosarcoma cell lines was down-regulated (Fig. 6D). As the functions of TNFRSF21 and necroptosis mediators were unknown in osteosarcoma, we further examined their expression levels. Western blot analysis revealed the levels of proteins of TNFRSF21, RIPK1, RIPK3 and MLKL were decreased (Fig. 6E). In brief, these data confirmed that necroptosis was inactive in osteosarcoma.

### TNFRSF21 inhibits osteosarcoma by promoting necroptosis

Firstly, we successfully established TNFRSF21-overexpressing cell lines (Supplemental Fig. 1). Then the impact of TNFRSF21 on osteosarcoma cells was detected.

**Fig. 7:**
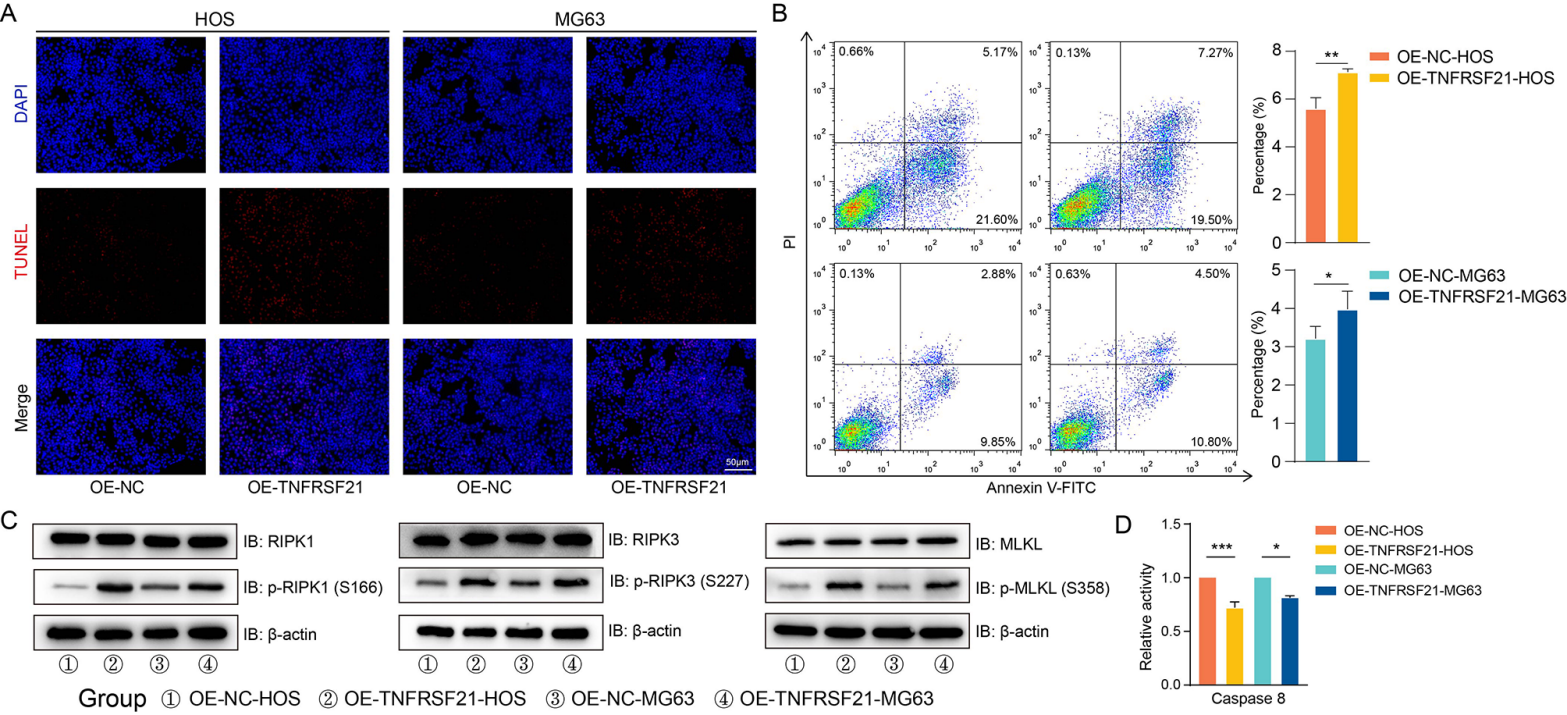
Inhibitory effect of TNFRSF21 on osteosarcoma. Subcutaneous tumors were dissected and photographed after 4 weeks, *P* value was calculated by 2-tailed Student t test (n = 6) (**A**). EdU staining (green fluorescence) on cell proliferation 2 days after the construction of osteosarcoma cell lines overexpressing TNFRSF21, nuclei were counterstained blue with DAPI (Scale bars, 50 μm) (**B**). Cell proliferation was detected by measuring the optical density in 450 nm, *P* value was calculated by 2-tailed Student t test (n = 4) (***, *P* < 0.001) (**C**). Representative images of colony formation assays in eighth day after the construction of osteosarcoma cell lines overexpressing TNFRSF21, *P* value was calculated by 2-tailed Student t test (n = 3) (***, *P* < 0.001) (**D**). Representative images of wound healing assays after 12 h of the gap formation (Scale bars, 500 μm) (**E**). Representative images of Transwell assays (migration and invasion) after 24 h incubation (Scale bars, 100 μm), *P* value was calculated by 2-tailed Student t test (n = 3) (*, *P* < 0.05; ***, *P* < 0.01; ***, *P* < 0.001) (**F**)

We explored the effect of TNFRSF21 overexpression on osteosarcoma growth in vivo. In Fig. 7A, TNFRSF21 overexpression resulted in a significant reduction in tumor volume and weight. As show in Fig. 7B and C, TNFRSF21 inhibited cell proliferation both in HOS and MG63 cell lines (*P* < 0.001). This phenomenon was further validated in colony form assay (Fig. 7D). Moreover, the migration and invasion of osteosarcoma cells were also attenuated (Fig. 7E and F).

**Fig. 8:**
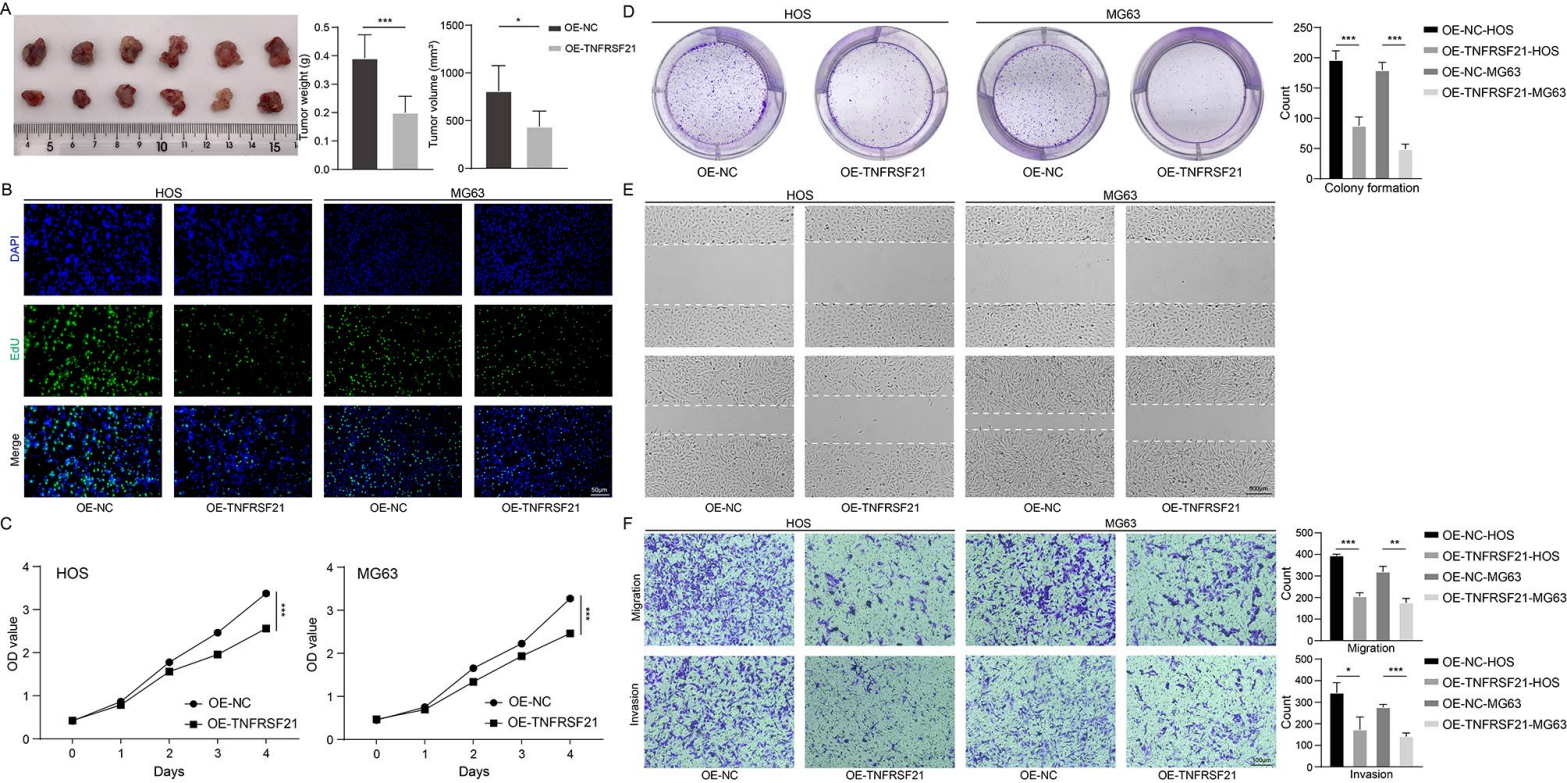
Overexpression of TNFRSF21 promoted necroptosis. TUNEL staining (red fluorescence) on cell apoptosis, nuclei were counterstained blue with DAPI (Scale bars, 50 μm) (**A**). Percentage of cells in the late stages of apoptosis was assessed via flow cytometry, *P* value was calculated by 2-tailed Student t test (n = 3)(**, *P* < 0.01; ***, *P* < 0.001;) (**B**). The phosphorylation levels of necroptosis mediators were detected by western blot (**C**). The activity of caspase 8 was assessed by measuring the optical density in 405 nm. Incremental activity of caspase 8 was expressed as a percentage of control, *P* value was calculated by 2-tailed Student t test (n = 3)(*, *P* < 0.05; ***, *P* < 0.001;) (**D**)

After TNFRSF21 overexpression, the proportion of apoptotic cells was increased (Fig. 8A). Taking this one step further, flow cytometry revealed the percentage of late apoptotic and secondary necrotic cells (FITC+/PI+) was higher (Fig. 8B).

To test the hypothesis that TNFRSF21 promoted necroptosis in osteosarcoma, we examined the phosphorylation levels of RIPK1, RIPK3 and MLKL [6]. Western blot analysis indicated the level of phospho-RIPK1 at Ser166 was increased, so did phospho-RIPK3 at Ser227 and phospho- MLKL at Ser358. And protein levels of RIPK1, RIPK3 and MLKL were not statistically altered (Fig. 8C). Furthermore, caspase 8 activity was significantly inhibited in cells with TNFRSF21 overexpression (Fig. 8D). Together, these data indicated TNFRSF21 inhibited cell proliferation and motility by activating necroptosis.

## Discussion

Cell death is of significant function in physiological and pathological process [40]. As a “hybridized product” of necrosis and apoptosis, the effect of necroptosis in diseases has been extensively reported. In a way, necroptosis is the “backup” of apoptosis [41]. Therefore, when apoptosis is blocked, it is very attractive to kill tumor cells by inducing necroptosis [42]. However, the role of necroptosis is not clear in osteosarcoma. In this study, we attempted to understand osteosarcoma in terms of necroptosis. After a series of analyses, a necroptosis prognostic signature and derived NS based on NRGs was successfully established. Our data showed this signature could effectively predict prognosis in osteosarcoma. Although the analysis of retrospective data yielded a valuable signature, prospective cohort studies are needed for further validation.

We also found that patients with more active necroptosis had a better prognosis. This protective effect might appear through the modification of tumor microenvironment by necroptosis. Because in Figs. 3E and 4B, we observed necroptosis had impact on immune infiltration and cell proliferation of osteosarcoma. In general, inflammatory response is weaker in apoptosis due to the intact plasma membrane [43]. But when necroptosis occurs, cell membrane is destroyed, the release of cellular contents and damage-associated molecular patterns often provokes a vigorous inflammatory response [5]. This is one of the most leading differences of necroptosis and apoptosis. This pro-inflammatory effect is also diminished when necroptosis is inhibited. In addition, RIPK3, one of the core mediators of necroptosis, has been demonstrated to promote the function of macrophages and dendritic cells [44]. RIPK1and RIPK3 are able to directly promote the priming of CD8^+^T cells and anti-tumor immune response [45, 46]. These pro-immunity effects exerted by these core mediators may be weakened on the condition of inactive necroptosis.

In contrast to the decreased level of immune infiltration, we found significantly higher tumor purity and stemness index in the high-score group. Increased stemness index indicated the gradual loss of differentiated phenotype and acquisition of progenitor-like, stem cell-like features in tumor cells [28]. At present, it is known that there are more cancer stem cells in malignant solid tumors, which can promote development and metastasis [47]. Furthermore, the proportion of tumor cells in the tumor tissue (namely tumor purity) increased to contribute to this process. Considering about that immune infiltration levels were significantly decreased in high-score group, we can make the following inference: osteosarcoma may “purify” itself by evading necroptosis, suppressing immune infiltration and dedifferentiating phenotypes. And this will lead to the progression of tumor and worse prognosis [48–51].

In 1998, G Pan et al. identified TNFRSF21 and performed its functional characterization [52]. They found TNFRSF21 induced cellular apoptosis. Later, a study reported that TNFRSF21 induced apoptosis by interacting with Bax [53]. There was another interesting phenomenon in this work: the inhibition of caspase 8 had no effect on TNFRSF21-induced apoptosis. According to the current knowledge, we recognize this phenomenon is one of the characteristics of necroptosis. There are some studies on the role of TNFRSF21 in tumors such as gastric cancer, pancreatic adenocarcinoma and glioma [54–56]. However, the specific mechanisms about how TNFRSF21 plays its role in necroptosis is not clearly elucidated. In this study, we demonstrated that TNFRSF21 acted as an inhibitory factor of osteosarcoma development by promoting necroptosis. Our data showed that osteosarcoma patients with high expression of TNFRSF21 had relatively higher survival rate. Overexpression of TNFRSF21 inhibited the growth and motility of osteosarcoma. More importantly, we determined that the phosphorylation levels of RIPK1/3 and MLKL increased and caspase 8 activity decreased upon TNFRSF21 overexpression. This suggested that TNFRSF21 activated necroptosis in osteosarcoma. However, the link between TNFRSF21 and immune cell in osteosarcoma needs to be further explored.

## Conclusions

This study established a necroptosis prognostic signature and derived NS based on data from TCGA and GEO. The signature serves as a stable and independent prognostic biomarker. Inhibition of necroptosis leads to increased tumor cell communication, decreased immune cell infiltration levels, and a worse prognosis for osteosarcoma. This study contributes to a deeper understanding of osteosarcoma through the lens of necroptosis. Additionally, our findings suggest that TNFRSF21 could serve as a treatment target through necroptosis, offering a novel approach for osteosarcoma treatment.

### Electronic supplementary material

Below is the link to the electronic supplementary material.

Supplemental figure 1: Construction of osteosarcoma cell lines overexpressing TNFRSF21, *P* value was calculated by 2-tailed Student t test (n = 3)(***, *P* < 0.001)

Supplemental Table 1: Demographic and clinical characteristics of patients

Supplemental Table 2: Primers used in the study

## Data Availability

The datasets analyzed during the current study are available from The Cancer Genome Atlas (TCGA) and the Gene Expression Omnibus (GSE39055, GSE21257 and GSE162454).
