# Supplementary figures and images for: Identification of TNFRSF21 as an inhibitory factor of osteosarcoma based on a necroptosis-related prognostic gene signature and molecular experiments
Source: Cancer Cell Int. 2024 Jan 6;24:14. doi: 10.1186/s12935-023-03198-w (PMC10770912; doi:10.1186/s12935-023-03198-w)

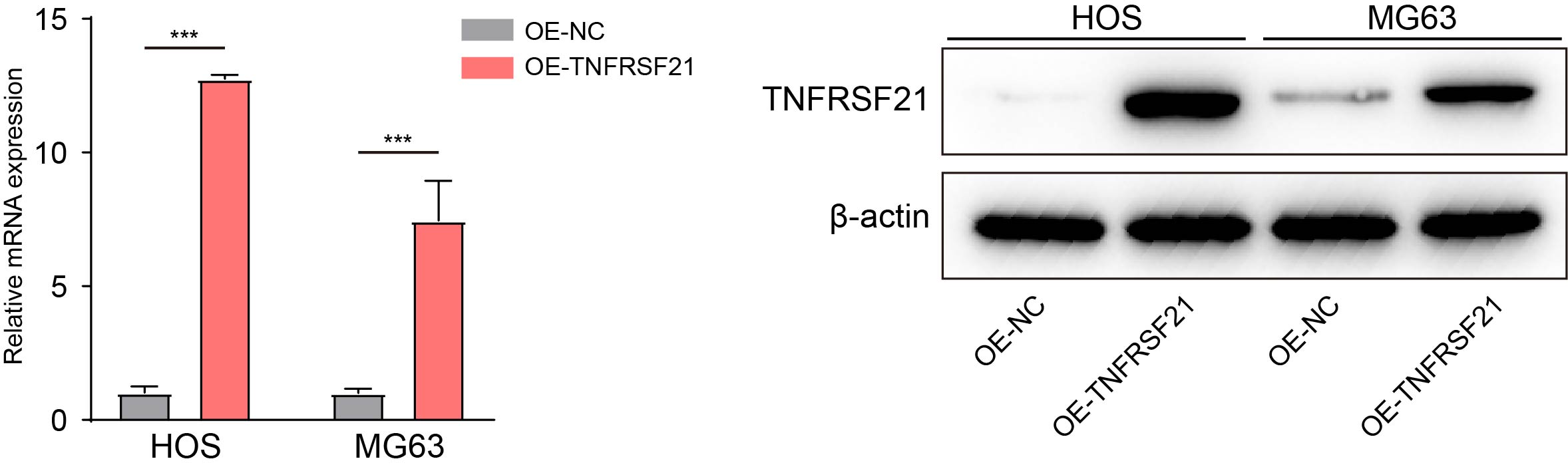

Supplement: Supplementary file 1 — Supplemental figure 1: Construction of osteosarcoma cell lines overexpressing TNFRSF21, P value was calculated by 2-tailed Student t test (n = 3)(***, P < 0.001) [file 12935_2023_3198_MOESM1_ESM.jpg]
