# Supplementary material for: Identification of TNFRSF21 as an inhibitory factor of osteosarcoma based on a necroptosis-related prognostic gene signature and molecular experiments
Source: Cancer Cell Int. 2024 Jan 6;24:14. doi: 10.1186/s12935-023-03198-w (PMC10770912; doi:10.1186/s12935-023-03198-w)
Supplement: Supplementary file 2 — Supplemental Table 1: Demographic and clinical characteristics of patients [file 12935_2023_3198_MOESM2_ESM.docx]

**Supplemental Table 1.** Demographic and clinical characteristics of patients.

| Characteristics | TCGA cohort |
| --- | --- |
| Sex (male/female) | 47/37 |
| Age (years) | 14.98±4.78 |
| Survival status (alive/dead) | 55/29 |
| Average survival time (months) | 76.21±68.81 |
| Metastasis at diagnosis (Yes/No/Unknown) | 3/14/70 |
