# Supplementary material for: Identification of TNFRSF21 as an inhibitory factor of osteosarcoma based on a necroptosis-related prognostic gene signature and molecular experiments
Source: Cancer Cell Int. 2024 Jan 6;24:14. doi: 10.1186/s12935-023-03198-w (PMC10770912; doi:10.1186/s12935-023-03198-w)
Supplement: Supplementary file 3 — Supplemental Table 2: Primers used in the study [file 12935_2023_3198_MOESM3_ESM.docx]

**Supplemental Table 2.** Primers used in the study.

| Gene symbol | Full name |  | Sequence (5’-3’) |
| --- | --- | --- | --- |
| CCL2 | C-C Motif Chemokine Ligand 2 | Forward | AATCAATGCCCCAGTCACCT |
|  |  | Reverse | CTTCTTTGGGACACTTGCTGC |
| FAP | Fibroblast Activation Protein Alpha | Forward | GGTCGCCTGTTGGGAGTAAA |
|  |  | Reverse | AGGAGACCACCAGAGAGCAT |
| HGF | Hepatocyte Growth Factor | Forward | CGGGGTAAAGACCTACAGGA |
|  |  | Reverse | AGCGTACCTCTGGATTGCTT |
| TNFRSF1A | TNF Receptor Superfamily Member 1A | Forward | GTTGTGCCTACCCCAGATTGA |
|  |  | Reverse | AAGCTCCCCCTCTTTTTCAGG |
| TNFRSF21 | TNF Receptor Superfamily Member 21 | Forward | TAGTCAACCACCAGCAAGGC |
|  |  | Reverse | AGCACAATCATCCAGGGCAA |
| RIPK1 | Receptor Interacting Serine/Threonine Kinase 1 | Forward | TAAGAAGAATGGCGGCACCC |
|  |  | Reverse | TTCTCTGTGGGCTTTGCGTT |
| RIPK3 | Receptor Interacting Serine/Threonine Kinase 3 | Forward | ACTCCCGGCTTAGAAGGACT |
|  |  | Reverse | TCCTTTACCGTGGAGACAGC |
| MLKL | Mixed Lineage Kinase Domain-Like | Forward | GACCAAGGAAAGAGGAGCGT |
|  |  | Reverse | GCTTCCTGTTCACGTCCTTG |
| β-actin | Actin Beta | Forward | CTCCATCCTGGCCTCGCTGT |
|  |  | Reverse | GCTGTCACCTTCACCGTTCC |
